# Predictors of treatment failure for non-severe childhood pneumonia in developing countries – systematic literature review and expert survey – the first step towards a community focused mHealth risk-assessment tool?

**DOI:** 10.1186/s12887-015-0392-x

**Published:** 2015-07-09

**Authors:** Eric D. McCollum, Carina King, Robert Hollowell, Janet Zhou, Tim Colbourn, Bejoy Nambiar, David Mukanga, Deborah C. Hay Burgess

**Affiliations:** Department of Pediatrics, Division of Pulmonology, Johns Hopkins School of Medicine, Baltimore, USA; Institute for Global Health, University College London, London, UK; The Boston Consulting Group, Boston, USA; Bill & Melinda Gates Foundation, Seattle, USA

**Keywords:** integrated Community Case Management (iCCM), Community health workers, Childhood pneumonia, Pediatrics, Developing countries, Predictors, Treatment failure, mHealth

## Abstract

**Background:**

Improved referral algorithms for children with non-severe pneumonia at the community level are desirable. We sought to identify predictors of oral antibiotic failure in children who fulfill the case definition of World Health Organization (WHO) non-severe pneumonia. Predictors of greatest interest were those not currently utilized in referral algorithms and feasible to obtain at the community level.

**Methods:**

We systematically reviewed prospective studies reporting independent predictors of oral antibiotic failure for children 2–59 months of age in resource-limited settings with WHO non-severe pneumonia (either fast breathing for age and/or lower chest wall indrawing without danger signs), with an emphasis on predictors not currently utilized for referral and reasonable for community health workers. We searched PubMed, Cochrane, and Embase and qualitatively analyzed publications from 1997–2014. To supplement the limited published evidence in this subject area we also surveyed respiratory experts.

**Results:**

Nine studies met criteria, seven of which were performed in south Asia. One eligible study occurred exclusively at the community level. Overall, oral antibiotic failure rates ranged between 7.8-22.9 %. Six studies found excess age-adjusted respiratory rate (either WHO-defined very fast breathing for age or 10–15 breaths/min faster than normal WHO age-adjusted thresholds) and four reported young age as predictive for oral antibiotic failure. Of the seven predictors identified by the expert panel, abnormal oxygen saturation and malnutrition were most highly favored per the panel’s rankings and comments.

**Conclusions:**

This review identified several candidate predictors of oral antibiotic failure not currently utilized in childhood pneumonia referral algorithms; excess age-specific respiratory rate, young age, abnormal oxygen saturation, and moderate malnutrition. However, the data was limited and there are clear evidence gaps; research in rural, low-resource settings with community health workers is needed.

**Electronic supplementary material:**

The online version of this article (doi:10.1186/s12887-015-0392-x) contains supplementary material, which is available to authorized users.

## Background

Healthcare providers in resource-limited countries use practical, standardized case management guidelines to diagnose pneumonia [1]. Although mortality reductions of 36 % have been attributed to standardized management, pneumonia remains the second leading cause of childhood death globally with nearly one million deaths annually [2]. Three-quarters of all deaths occur in Africa and Asia, and it is estimated that a majority of all childhood pneumonia deaths occur outside of the hospital [3, 4]. Incomplete antibiotic coverage and care-seeking delays negatively influence pneumonia outcomes in low-resource settings and improvements have been inconsistent and difficult to sustain [4].

The World Health Organization (WHO) recommends expansion of basic healthcare beyond traditional facilities to community health workers (CHWs). This strategy, called ‘integrated Community Case Management’ (iCCM), includes childhood pneumonia care [5]. iCCM can address gaps in antibiotic access and care seeking delays by providing antibiotics earlier in a pneumonia illness when the disease is more responsive to treatment [5, 6]. Likewise, iCCM can permit earlier referral if treatment fails - defined as disease persistence or progression despite antibiotics. Evidence from Zambia and Pakistan has demonstrated improved childhood pneumonia antibiotic coverage and reduced treatment failure with iCCM [7–9]. As a result, iCCM expansion is a key strategy against childhood pneumonia [5, 6].

Despite iCCM’s potential there are challenges, such as sustaining quality of care. CHWs receive limited training and are difficult to supervise in remote areas [5, 6]; consistent supervision and retraining is likely important for successful iCCM pneumonia management to ensure standardized recording of subjective clinical danger signs [10]. Strengthening CHW decision-making and identification of higher-risk patients, including those that do not meet current referral criteria, could reduce incorrect pneumonia diagnosis, antibiotic prescription, and referrals. Mobile health, or *mHealth*, may help to address these weaknesses [11]. mHealth utilizes user-friendly software compatible with mobile phones for healthcare delivery and capitalizes on mobile networks with wide coverage and usage in developing countries [11]. New mHealth applications that provide an array of performance support for CHWs are in development [12, 13].

An iCCM mHealth mobile phone application could assist CHWs to identify two groups of children with pneumonia; (1) those who meet current referral criteria but are mis-classified, and (2) those who do not meet current referral criteria but are still at greater risk for treatment failure based upon their initial clinical assessment. Both groups of children could then be referred for more appropriate treatment or be flagged for closer community-based monitoring. An improved referral mHealth tool could provide the result as a simple audio recommendation translated into the preferred local language, allowing both the CHW and caregiver to hear the recommendation. Facilitating objective decision-making by CHWs with a mHealth tool could complement existing guidelines to expand appropriate, timely antibiotic coverage and further reduce life-threatening treatment delays.

This systematic review and expert opinion survey aimed to identify predictors of WHO non-severe pneumonia treatment failure that are not currently used in referral algorithms and are also realistic for CHWs. The ultimate aim of a prediction algorithm would be to facilitate improved referral pathways originating from the community level, leading both to improved antibiotic stewardship and decreased morbidity and mortality.

## Methods

### Systematic review

We conducted a systematic review in accordance with PRIMSA (see Additional file 1) [14]. All studies from January 1, 1997 - June 25, 2014 listed in PubMed, Cochrane Central Register of Controlled Trials, and Embase were eligible. The following search terms were used: pneumonia; predictors or risk factors or prognostic; treatment failure or death; and Africa or Asia or developing country. These were combined with the following search filters: clinical trial, humans, and infant or preschool ages. No language restrictions were applied. We conducted the search on June 25, 2014. We also conducted a supplementary, non-systematic search that did not filter for geographical region. Please see Additional file 2 for these findings.

**Fig. 1:**
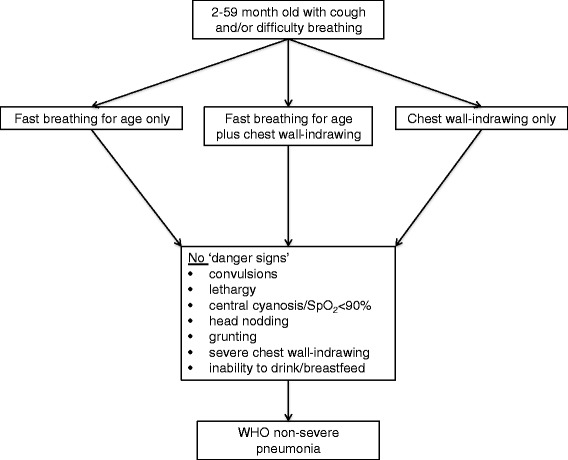
2013 World Health Organization non-severe pneumonia case definition

Titles and abstracts were reviewed twice and those which both reviewers agreed were relevant underwent full text review. Reference lists of reviewed manuscripts were screened for additional studies. Eligibility criteria included: 1) Study design: Prospective observational studies and clinical trials. 2) Participants: Aged 2–59 months treated with *oral* antibiotics for WHO-defined non-severe pneumonia per the 2013 revised WHO guidelines (Fig. 1) [15]. 3) Data collection dates: 1997 or more recent. The current WHO guidelines were formally disseminated in 1997 [1]. 4) Geographic regions: Africa and/or Asia, representing the highest burden of disease. 5) Outcome: *A priori* defined treatment failure, with statistical analysis for independent baseline clinical predictors of treatment failure.

We used a structured data extraction tool to describe key data from the eligible studies including: region, study design, primary outcome, data collection dates, enrollment criteria, treatment failure definition, sample size, age range of participants, treatment failure rate, and independent predictors of treatment failure. The Cochrane Risk of Bias tool was used to qualitatively assess the risk of bias in individual studies [16].

### Expert opinion

We complemented the systematic literature review by surveying childhood pneumonia experts. The experts were all healthcare professionals experienced in childhood pneumonia in the African or Asian region across hospital and community care settings, many with research experience [17]. We used a modified Delphi consensus process, administering two separate structured phone interviews and a final electronic survey to 13 experts, concluding when a general consensus was reached (see Additional file 3 for interview materials). Participants were asked to assess the potential role for a pneumonia treatment prognostic, including where in the health system (the home, a community health clinic, or the hospital) a prognostic would be best utilized.

The literature search provided the initial prognostic indicators to test with interviewees, using WHO definitions [15]. The panel was allowed to adjust these indicators, as well as the definitions of them and we encouraged the panel to use their own knowledge and experience. Fever was modified to an axillary temperature >38.0 °C, high blood lactate levels to >2 mmol/l, and low weight for age as a standardized z score < −2. The definition of ‘human immunodeficiency virus (HIV)-affected’ included HIV-exposed children (i.e. born to an HIV-infected mother but not HIV tested *or* previously tested HIV-negative with ongoing breastfeeding) or HIV-infected per standard guidelines [18].

The experts qualitatively and quantitatively estimated each predictor’s potential value. Qualitative estimates of each prognostic indicator’s value were allocated using an ordinal scale (0 = unfavorable, 1 = minimally favorable, 2 = favorable, 3 = highly favorable), along with any comments regarding each indicator’s likely performance and feasibility. Panel members provided quantitative estimates by assigning a childhood pneumonia mortality rate to community oral *or* parenteral hospital antibiotic treatment.

An anonymous summary of panel answers were provided to interviewees after the first interview and each expert was encouraged to revise their answers after considering the panel’s overall results. For the final survey, the expert opinion results were rephrased into binary yes/no questions to determine a final consensus. This research was approved by the Johns Hopkins Institutional Review Board (IRB00047406) and written consent was obtained from survey participants.

## Results

### Systematic review

**Fig. 2:**
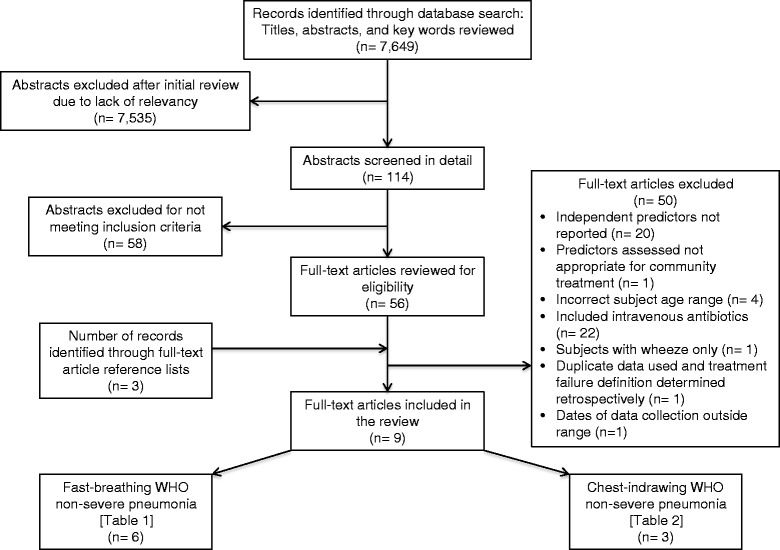
Flow diagram of study identification and selection

Our search yielded 7,649 studies; of these, 59 manuscripts were retrieved for full text review. Nine studies met all eligibility criteria, including quality control, and were qualitatively analyzed (Fig. 2). We found no publications that included children with fast breathing *and* lower chest indrawing (LCI)-defined pneumonia together, probably because the WHO guidelines were only recently revised and disseminated in 2013.

Table 1 summarizes the six studies reporting predictors of oral antibiotic failure in fast breathing non-severe pneumonia [19–24]. All studies were from developing countries in south Asia. Five of the six studies included amoxicillin treatment and only one included community clinics. Five studies defined treatment failure as either a persistence of fast breathing after 3–5 days or disease progression (development of LCI or danger signs) between 1–5 days after antibiotic initiation. One study included peripheral oxygen saturation (SpO_2_) less than 90 % in the definition of treatment failure. The proportion of children that failed oral antibiotic treatment ranged from 7.8 % to 22.9 %. Three studies identified baseline excess age-specific respiratory rate, and two studies found history of difficulty breathing, age, and illness duration of ≥3 days as independent predictors of oral antibiotic failure.

**Table 1:** Independent predictors of oral antibiotic failure in fast breathing WHO non-severe childhood pneumonia^a^

Study overview		Awasthi S et al.	CATCH UP	MASCOT	Agarwal G et al.	Noorani QA et al.	Hazir T et al.
Region (country)		Asia (India)	Asia (Pakistan)	Asia (Pakistan)	Asia (India)	Asia (Pakistan)	Asia (Pakistan)
Data collection dates		2004–2006	1998–1999	1999–2001	2000–2002	2000–2001	2005–2008
Enrollment criteria		2–59 months old	2–59 months old	2–59 months of age	2–59 months of age	2–59 months of age	2–59 months of age
Study design		Cluster randomized study	Randomized multicenter equivalency study	Randomized multicenter equivalency study	Randomized multicenter equivalency study	Multicenter observational study	Randomized multicenter equivalency study
	Intervention arm	Oral amoxicillin 31–51 mg/kg/day for 3 days	Oral amoxicillin 50 mg/kg/day for 5 days	Oral amoxicillin 45 mg/kg/day for 3 days	Oral amoxicillin 31–54 mg/kg/day for 3 days	-	Placebo for 3 days
	Control arm (or standard of care)	Oral trimethoprim (7–11 mg/kg/day)-sulfamethoxazole for 5 days	Oral trimethoprim (8 mg/kg/day)-sulfamethoxazole for 5 days	Oral amoxicillin 45 mg/kg/day for 5 days	Oral amoxicillin 31–54 mg/kg/day for 5 days	Oral trimethoprim (8 mg/kg/day)-sulfamethoxazole for 5 days	Oral amoxicillin 45 mg/kg/day for 3 days
	Blinding	No	Yes	Yes	Yes	-	Yes
	Study site types	Health centers	Tertiary care and secondary-level hospitals, and health center	Tertiary care and secondary-level hospitals	Tertiary care hospitals	Health centers	Tertiary care hospitals
	Number of sites	14	8	7	7	14	4
	Primary outcome	Treatment failure	Equivalency	Equivalency	Equivalency	Treatment failure	Equivalency
Treatment failure definition							
	Day of assignment	Day 3 (intervention); Day 5 (control)	Days 3 to 5	Day 5	Days 3 to 5	Day 5	Day 3
	Respiratory rate^a^	Yes, elevated for age (days 3–5)	Yes, +/−5 breaths/min or higher versus enrollment (days 3–5)	Yes, elevated for age (days 3–5)	Yes, elevated for age (days 3–5)	Yes, +/−5 breaths/min or higher versus enrollment (days 3–5)	No
	Fever >38 °C *and* LCI	No	No	No	No	No	No
	Fever >38 °C	Yes (days 3–5)	No	No	No	No	No
	LCI	Yes (days 1–5)	Yes (days 3–5)	Yes (days 1–5)	Yes (days 1–5)	Yes (days 1–5)	Yes (days 1–3)
	Convulsions	Yes (days 1–5)	Yes (days 3–5)	Not specified	Yes (days 1–5)	Not specified	Yes (days 1–3)
	Abnormally sleepy	Yes (days 1–5)	Yes (days 3–5)	Not specified	Yes (days 1–5)	Not specified	Yes (days 1–3)
	Inability to drink	Yes (days 1–5)	Yes (days 3–5)	Not specified	Yes (days 1–5)	Not specified	Yes (days 1–3)
	Stridor in calm child	Yes (days 1–5)	Yes (days 3–5)	No	No	No	Yes (days 1–3)
	Malnutrition	No	Yes (days 3–5)	No	No	No	Yes (days 1–3)
	Cyanosis	Yes (days 1–5)	No	Yes	No	Yes	No
	SpO_2_	No	No	No	Yes, <90 % (on day 3 only)	No	No
	Antibiotic change	No	Yes (days 3–5)	Yes (days 1–5)	No	Yes (days 1–5)	No
	Hospitalization	No	Yes (days 1–5)	No	No	No	No
	Lost to follow-up	Yes (days 3–5)	Yes (days 3–5)	No	Yes (days 1–5)	Yes (days 5–11)	No
	Study withdrawal	Yes (days 1–5)	No	No	Yes (days 1–5)	No	No
	Death	Yes (days 1–14)	Yes (days 1–5)	Yes (days 1–5)	Yes (days 1–5)	Yes (days 1–5)	Yes (days 1–5)
Study participants and description							
Sample size^b^		2009	1459	1953	2188	944	873
Age 2–11 months		594/2009 (29.6 %)	732/1459 (50.2 %)	1051/1953 (53.8 %)	954/2188 (43.6 %)	334/944 (35.4 %)	573/873 (65.6 %)
Age 12–59 months		1415/2009 (70.4 %)	727/1459 (49.8 %)	902/1953 (46.2 %)	1234/2188 (56.4 %)	610/944 (64.6 %)	300/873 (34.4 %)
Treatment failure rate^b^		234/2009 (11.6 %)	256/1459 (17.5 %)	364/1953 (22.9 %)	225/2188 (10.3 %)	110/944 (11.6 %)	68/873 (7.8 %)
Independent predictors of treatment failure OR (95 % CI)^c,d^		Diarrhea, 1.65 (1.24–2.19)	History of difficulty breathing, 1.61 (1.13–2.15)	Excess respiratory rate >10 breaths/min for age^a^, 1.4 (1.1–1.9)	Excess respiratory rate >10 breaths/min for age^a^, 2.89 (1.83–4.55)	Excess respiratory rate ≥15 breaths/min for age^a^, 2.0 (1.2–3.4)	History of difficulty breathing, 2.86 (1.29–7.23)
			Age 12–59 months, 1.5 (1.12–1.91)	Antibiotic non-adherence, 4.5 (95 % CI 2.9–7.0)	Antibiotic non-adherence, 11.57 (7.4–18.0	Wheeze on examination, 1.7 (1.1–2.6)	Temperature >37.5 °C at enrollment, 1.99 (1.37–2.90)
			Illness >3 days, 1.4 (1.03–1.8)	Illness ≥3 days, 1.7 (1.3–2.1)	RSV, 1.95 (1.0–3.8)		
				Age 2–11 months, 1.7 (1.1–2.1)			
				Persistent vomiting, 1.4 (1.0–2.0)			

Serious adverse event or drug reaction, and new comorbid condition were not considered in the treatment failure definitions for any of the studies. WHO: World Health Organization; LCI: lower chest wall indrawing; SpO_2_: peripheral oxygen saturation; OR: odds ratio; CI: confidence interval; RSV: human respiratory syncytial virus

^a^Respiratory rate norms: <50 breaths/min for ages 2–11 months; <40 breaths/min for ages 12–59 months

^b^If primary outcome of trial was equivalency or no difference was found then intervention and control arm data was combined

^c^Multivariate logistic regression modeling was performed to determine independent predictors of treatment failure in all included studies. CATCHUP trial modeled age, history of difficulty breathing, duration of illness, and respiratory rate. The models were not reported for the MASCOT trial, Agarwal G et al., Noorani QA et al., Awasthi S et al., and Hazir T et al

^d^For Hazir T et al. multivariate logistic regression modeling was performed on cumulative treatment failure data from day 5 although primary outcome data was analyzed from day 3 treatment failure data

Table 2 summarizes the three studies reporting independent predictors of oral antibiotic failure in LCI non-severe pneumonia [25–27]. Two studies were multi-country with sites in Africa. In two of the three studies, treatment failure was assigned on day six after antibiotic initiation. Treatment failure rates for the three studies ranged from 8.1 % to 19.3 %. All studies found young age and very fast breathing at baseline to be independent predictors of treatment failure. One study collected SpO_2_ measurements and found low baseline SpO_2_ to predictive; another study found malnutrition to be predictive of treatment failure.

**Table 2:** Independent predictors of oral antibiotic failure in chest indrawing WHO non-severe childhood pneumonia

Study overview		Addo-Yobo E, Chisaka N et al.	Hazir T, Fox LM et al.	Addo-Yobo E, Anh DD et al.
Region (countries)		Africa (Ghana, South Africa, Zambia) Asia (India, Pakistan, Vietnam) South America (Columbia, Mexico)	Pakistan (Asia)	Africa (Egypt, Ghana) Asia (Bangladesh, Vietnam)
Data collection dates		1999–2002	2005–2006	2005–2008
Enrollment criteria		3–59 months	3–59 months	3–59 months
Study design		Randomized multicenter equivalency study	Randomized multicenter equivalency study	Multicenter observational study
Intervention arm		Oral amoxicillin 45 mg/kg/day for 5 days	Oral amoxicillin 80–90 mg/kg/day for 5 days	-
Control arm (or standard of care)		Intravenous 200,000 IU penicillin G for 2 days then oral amoxicillin 45 mg/kg/day for 3 days (total 5 days)	Intravenous ampicillin 100 mg/kg/day for 2 days then oral amoxicillin for 3 days (total 5 days)	Amoxicillin 80–90 mg/kg/day for 5 days
Blinding		No	No	-
Study site type(s)		Pediatric department of tertiary care hospitals	Pediatric department of tertiary care hospitals	Pediatric department of tertiary care and second-level hospitals, health centers
Number of study sites		9	7	5
Primary outcome		Equivalence	Equivalence	Treatment failure
Treatment failure definition				
Day of assignment		Day 3	Day 6	Day 6
Respiratory rate^a^		No	No	No
Fever >38 °C *and* LCI		No	Yes (days 3–6)	Yes (day 3)
Fever >38 °C		No	Yes (day 6)	Yes (day 6)
LCI		Yes (day 3)	Yes (day 6)	Yes (day 6)
Convulsions		Yes (days 1–3)	Yes (days 1–6)	Yes (days 1–6)
Abnormally sleepy		Yes (days 1–3)	Yes (days 1–6)	Yes (days 1–6)
Inability to drink		Yes (days 1–3)	Yes (days 1–6)	Yes (days 1–6)
Stridor in calm child		No	No	No
Malnutrition		No	No	No
Cyanosis		Yes (days 1–3)	Yes (days 1–6)	Yes (days 1–6)
SpO_2_		<80 % at sea-level or <75 % below sea-level (days 1–3)	No	No
Antibiotic change		Yes (days 1–3)	No	Yes (days 1–6)
Hospitalization		No	Yes, if related to pneumonia (days 1–6)	No
Serious drug reaction		Yes (days 1–3)	No	Yes (days 1–6)
Serious adverse event		No	Yes (days 1–6)	No
New comorbid condition		Yes (days 1–3)	Yes, if required antibiotic (days 1–6)	Yes (days 1–6)
Lost to follow-up		Yes (days 1–3)	Yes (days 1–6)	Yes (day 6)
Study withdrawal		Yes (days 1–3)	Yes (days 1–6)	No
Death		Yes (days 1–3)	Yes (days 1–14)	Yes (days 1–6)
Study participants and description				
Sample size^b^		1702	2037	823
Age:	3–11 months	1045/1669 (62.6 %)	1311/2037 (64.4 %)	562/873 (64.4 %)
	12–59 months	624/1669 (37.4 %)	726/2037 (35.6 %)	310/873 (35.5 %)
Treatment failure rate^c^		328/1702 (19.3 %)	164/2037 (8.1 %)	76/823 (9.2 %)
Independent predictors of treatment failure^c^ OR/RR (95 % CI)		Age 3–11 months, 2.72 OR (95 % CI 1.95–3.79)	Age 3–5 months, 3.22 OR, (95 % CI 1.87–5.52)	Age 3–5 months, 1.96 RR (95 % CI 1.09–3.51)
		Very fast breathing, 1.94 OR (95 % CI 1.42–2.65)^d^	Very fast breathing, 1.65 OR (95 % CI 1.07–2.57)^d^	Very fast breathing, 12.5 RR (95 % CI 1.74–89.1)^d^
		SpO_2_ < 90 %, 2.11 OR (95 % CI 1.6–2.78)	Weight for age z score <−2, 1.79 OR (95 % CI 1.23–2.60)	

*WHO*: World Health Organization; *IU*: International Units; *LCI*: lower chest wall indrawing; *SpO*_*2*_: peripheral oxygen saturation; *OR*: odds ratio; *RR*: relative risk; *CI*, confidence interval

^a^Respiratory rate norms: <50 breaths/min for ages 2–11 months; <40 breaths/min for ages 12–59 months

^b^If primary outcome of trial was equivalency then intervention and control arm data was combined

^c^Multivariate logistic regression modeling was used by Addo-Yobo E, Chisaka N et al. and Hazir T while log-linear regression modeling was used by Fox LM et al. Addo-Yobo E, Chisaka N et al. modeled age, very fast breathing, hypoxemia, and amoxicillin. Hazir T, Fox LM et al. modeled sex, age, breastfeeding, weight-for-age z score <−2, very fast breathing, and treatment group (home vs hospital). Addo-Yobo E, Anh DD et al. modeled sex, age, and respiratory rate

^d^Very fast breathing defined as ≥70 breaths/min for ages 3–11 months and ≥60 breaths/min for ages 12–59 months

### Expert opinion

Of the 13 experts interviewed, eight reported experience in Africa, four in Asia, and one in both. All interviewees agreed that the development of a pneumonia treatment prognostic could significantly advance childhood pneumonia care and that the community-level was the most attractive setting for implementation.

The panel qualitatively evaluated seven prognostic indicators for performance and feasibility at the community level (Table 3). Abnormal SpO_2_ and malnutrition were ranked highest while fever and high blood lactate levels were lowest. Abnormal SpO_2_ was judged to be objective, although the panel identified the need for a robust, precise, low-cost pulse oximeter for young children. Malnutrition was also valued by the experts as it is likely to have a high positive predictive value and the feasibility of reliable malnutrition screening was attractive, with tools such as tapes and scales being simple, accurate, and relatively inexpensive.

**Table 3:** Expert panel qualitative assessments of potential prognostic indicators

Prognostic indicator	Overall favorability	Selected performance comments	Selected feasibility comments
Fever	Unfavorable	Low specificity for identifying children who will not fail treatment	High feasibility since already implemented and measurement tools simple
Rapid breathing	Minimally favorable	Normal range highly variable	Measurement tools inaccurate
		Low sensitivity for hypoxemic children	Low feasibility with respect to sequential monitoring of respiratory rates over time
		Performance profile improves with sequential monitoring of respiratory rates over time	
Lower chest wall indrawing	Favorable	Low specificity for identifying children who will not fail treatment	Subjective sign with variable inter-provider agreement levels
Abnormal oxygen saturation	Highly favorable	High specificity for identifying children who will not fail treatment	Objective measurement but inter-provider agreement levels unknown
		Later indicator decreases sensitivity for identifying children who will fail treatment at the community-level	Robust, precise, low-cost point-of-care instrument needed
			Likely costly to implement and sustain
High blood lactate levels	Unfavorable	Low specificity for identifying children who will not fail treatment	Point-of-care tool appropriate for community use not available, likely costly
		Very late indicator decreases sensitivity for identifying children who will fail treatment at the community-level	
Moderate malnutrition	Highly favorable	Mid-range sensitivity and specificity which likely improves greatly in combination with other indicators	High feasibility since measurement tools simple and accurate
		High positive predictive value	
HIV-affected	Minimally favorable	Geographically limited in relevancy (e.g. primarily in eastern and southern Africa)	Low feasibility since difficult to obtain HIV status at community-level (e.g. stigma)
		High positive predictive value	

*HIV*: Human Immunodeficiency Virus

The panel’s quantitative performance estimates for each prognostic indicator are presented in Table 4. Regardless of setting, children that are malnourished, HIV-affected or have an abnormal SpO_2_, were estimated to be at the highest mortality risk by the panel. Estimated median mortality rates of community treatment with amoxicillin for children with abnormal SpO_2_, malnutrition, or an HIV-affected status ranged from 15.5–20 %.

**Table 4:** Expert panel quantitative assessments of potential prognostic indicators

Identified prognostic indicator^a^	Mortality rate according to location of care received if prognostic indictor positive	
	Community treatment with oral amoxicillin	Hospital treatment with parenteral antibiotics
Fever	0.5 % (0.5–1.0)	0.1 % (0.1–0.2)
Rapid breathing	1.0 % (1.0–2.0)	0.2 % (0.1–0.5)
Lower chest indrawing	5.8 % (5.1–7.4)	1.0 % (0.6–1.0)
Abnormal SpO_2_	15.5 % (11.3–23.0)	6.3 % (5.0–7.5)
Moderate malnutrition	20.0 % (18.5–25.0)	10.5 % (5.0–12.8)
HIV-infected or HIV-exposed status	16.5 % (15.0–20.0)	7.5 % (6.1–8.6)

*IQR*: interquartile range; *SpO*_*2*_: peripheral oxygen saturation

^a^Lactate not assessed quantitatively

## Discussion

WHO guidelines standardize pneumonia management in low-resource settings and stratify pneumonia severity according to the presence of severe clinical features [1, 15]. Currently, these guidelines do not further categorize non-severe pneumonia patients according to their treatment failure risk even though some children with non-severe disease have failure rates as high as 23 % [20]. Given the increased decentralization of pneumonia care to CHWs, improving baseline risk stratification with objective clinical measures may be an important next step for reducing pneumonia morbidity. To assist in algorithm development, we performed a systematic review and surveyed experts to identify predictors of oral antibiotic failure in children that fit the WHO case definition for non-severe pneumonia (2013 guidelines) [15]. We placed the greatest emphasis on predictors not currently incorporated into referral algorithms and those that were also measureable by CHWs. From the literature and expert review, we found some agreement for excess age-specific respiratory rate, abnormal baseline SpO_2_ and moderate malnutrition as important predictors of treatment failure. However, the literature was limited and highlighted several key evidence gaps, and there were notable differences between those predictors supported by the literature and those favored by the experts.

Our systematic review and expert survey yielded several interesting comparisons. The panel strongly favored abnormal SpO_2_ as a predictor, however, SpO_2_ was evaluated in only two studies [21, 25], none of which examined SpO_2_ at community clinics or assessed less severe, but still abnormal SpO_2_ ranges (90–94 %). Similarly, malnutrition was highlighted by the panel but was only found to be predictive for oral antibiotic failure in one study conducted at tertiary hospital clinics [26]. Milder degrees of malnutrition were not evaluated, and the experts defined malnutrition in the moderate range. Although young age was found to be predictive in multiple studies [20, 25–27], the experts did not recommend young age as a candidate predictor. *Haemophilus influenzae* type B (HiB) vaccine or pneumococcal conjugate vaccine (PCV) status were not examined as predictors in our systematic review since most studies were performed before introduction of these vaccines, but it was also not recommended by the panel. These differences between the published literature and expert panel may in part reflect the panel’s emphasis on personal experience to guide their answers and the overall lack of publications meeting eligibility criteria. In addition, a majority of the panel worked in Africa while in contrast the published literature was heavily represented by Asia.

The systematic review suggested that fast breathing and LCI pneumonia may be distinct groups with different drivers and predictors of failure. Our review of the literature consistently found excess age-specific respiratory rate as an important predictor in both LCI- and fast breathing non-severe pneumonia [20–22, 25–27], but abnormal baseline SpO_2_ and young age were clearer predictors of treatment failure in children solely with LCI [25–27]. In those studies looking at fast-breathing defined pneumonia, the independent predictors were less consistent and included persistent vomiting, diarrhea, wheeze, human respiratory syncytial virus infection and history of difficult breathing; these may suggest a primarily viral cause of illness. Other studies support the notion that a low proportion of fast breathing pneumonia cases may be due to bacterial pneumonia. Hazir and colleagues reported that only 1.7 % of Pakistani children with fast breathing pneumonia had a radiographic consolidation consistent with a bacterial etiology [28], and the same group also published findings of equivalency when fast breathing cases were treated with either placebo or amoxicillin [23]. At this time listening for wheeze with a stethoscope or testing for respiratory syncytial virus are not feasible for routine CHW care. The results from this systematic review should be interpreted within this context; LCI and fast breathing pneumonia may differ etiologically and the published literature on this subject has primarily focused on fast breathing pneumonia (only three studies meeting eligibility criteria for this work included LCI pneumonia).

Our systematic review found an overall paucity of community-level data, potentially limited by publication bias. However, we also found other important evidence gaps. Firstly, it is likely that predictors will differ between African and Asian populations, in part because HIV is endemic in southern Africa and a key driver of treatment unresponsive pneumonia [29]. However, our systematic review demonstrated that African data is limited, with only two studies including data from African countries. These studies did not explore region as a predictor of treatment failure, and did not report adjusting for region in their multivariate models. The experts also did not address this evidence gap since they did not explicitly factor regional differences into their responses, although the majority of experts worked in Africa and this is likely to have influenced their answers. The development of a sound prognostic algorithm for the southern African region that accounts for HIV status may be challenging since HIV stigma persists, particularly in more rural, intimate community settings where anonymous testing is difficult to achieve [30]. Second, it is likely that pneumonia epidemiology, and thus treatment failure predictors, will shift after HiB vaccine and PCV introduction. However, data from developing countries after full vaccine introduction is only more recently becoming available [31]. Due to these limitations in the literature, the findings from the literature review need to be interpreted cautiously.

Although validated prognostic models that identify higher risk pneumonia patients exist, only two studies (both secondary analyses from the Amoxicillin Penicillin Pneumonia International Study (APPIS) [25]) present predictive models for children who fulfill the LCI non-severe pneumonia case definition [32, 33]. Mamtani et al. validated a clinical tool for predicting oral amoxicillin failure in children with LCI pneumonia and no danger signs, using the child’s age and excess age-specific respiratory rate after 24 h of treatment to stratify patients’ risk [32]. Fu et al. predicted the risk of oral amoxicillin treatment failure after 48 h, with the best performing model including a second clinical evaluation after 12 h of treatment and SpO_2_ [33]. However both studies likely reflect different pneumonia epidemiology due to increasing HiB and PCV availability and current treatment failure definitions have been simplified [26, 27]. The feasibility of using models that rely on a single patient observation for an extended period or multiple clinical observations will need careful assessment at the CHW level.

At this time more evidence is needed to demonstrate effective use of pulse oximetry by CHWs, whether milder degrees of altitude-adjusted hypoxemia (90–94 %) are predictive of treatment failure, and if pulse oximetry can be cost-effective at the community level in low-resource countries where equipment maintenance can be challenging. Several low-cost respiratory rate counters, pulse oximeters and combination devices have recently been developed or are in development for low-resource settings [34–36]; there is an on-going evaluation of the clinical performance, usability, and acceptability of these devices currently underway by Malaria Consortium (D. Hay Burgess, personal communication).

## Conclusion

In conclusion, we found that young age, excess age-specific respiratory rate, abnormal baseline SpO_2_ and moderate malnutrition should be carefully considered for an automated mHealth iCCM tool for predicting oral antibiotic failure in children with pneumonia. However, the evidence is currently limited. Additional formative research to identify antibiotic failure predictors is needed at the community-level, in HIV and non-HIV endemic regions, with and without malaria, and that have introduced the HiB vaccine and PCV. Modeling patient and health system-level consequences of a prognostic, along with a cost analysis of device scale-up, are important next steps.

## Additional files

Additional file 1: PRISMA checklist. ᅟ Additional file 2: Predicting treatment failure in developed countries. ᅟ Additional file 3: Expert panel interview materials. ᅟ

## Abbreviations

WHO: World Health Organization
iCCM: integrated Community Case Management
CHW: Community health worker
mHealth: Mobile health
HIV: Human immunodeficiency virus
LCI: Lower chest indrawing
SpO_2_: Peripheral oxygen saturation
HiB: *Haemophilus influenzae* type B
PCV: Pneumococcal conjugate vaccine
