# Supplementary material for: Predictors of treatment failure for non-severe childhood pneumonia in developing countries – systematic literature review and expert survey – the first step towards a community focused mHealth risk-assessment tool?
Source: BMC Pediatr. 2015 Jul 9;15:74. doi: 10.1186/s12887-015-0392-x (PMC4496936; doi:10.1186/s12887-015-0392-x)
Supplement: Additional file 2: Predicting treatment failure in developed countries. — ᅟ [file 12887_2015_392_MOESM2_ESM.docx]

**Additional file 1: Predicting treatment failure in developed countries**

We used the same search terms to look for studies conducted in developed countries as a comparison for predictors identified in from African and Asian studies. While we found several scores for assessing community-acquired pneumonia (CAP) severity, treatment and risk of death (e.g. Pneumonia Severity Index [[1](#_ENREF_1)], and CURB-65 [[2](#_ENREF_2)]), these were developed and validated in adults [[3-7](#_ENREF_3)]. There were also several papers investigating biological markers as predictors for poor outcomes, such as cortisol [[8](#_ENREF_8)], C-reactive protein and procalcitonin [[9-11](#_ENREF_9)], and prohormones [[12](#_ENREF_12)]. However, again these focused on hospitalized adult population. We did not find any published articles which matched our inclusion criteria for predictors of oral antibiotic treatment failure in pediatric pneumonia in developed countries, based on the WHO diagnosis criteria.

1. Fine, M.J., et al., *A Prediction Rule to Identify Low-Risk Patients with Community-Acquired Pneumonia.* New England Journal of Medicine, 1997. **336**(4): p. 243-250.

2. Lim, W.S., et al., *Defining community acquired pneumonia severity on presentation to hospital: an international derivation and validation study.* Thorax, 2003. **58**(5): p. 377-382.

3. Spindler, C. and A. Ortqvist, *Prognostic score systems and community-acquired bacteraemic pneumococcal pneumonia.* Eur Respir J, 2006. **28**(4): p. 816-23.

4. Valencia, M., et al., *Pneumonia severity index class v patients with community-acquired pneumonia: characteristics, outcomes, and value of severity scores.* Chest, 2007. **132**(2): p. 515-22.

5. Kothe, H., et al., *Outcome of community-acquired pneumonia: influence of age, residence status and antimicrobial treatment.* Eur Respir J, 2008. **32**(1): p. 139-46.

6. Schuetz, P., et al., *Predicting mortality with pneumonia severity scores: importance of model recalibration to local settings.* Epidemiology and Infection, 2008. **136**(12): p. 1628-37.

7. Yandiola, P.P., et al., *Prospective comparison of severity scores for predicting clinically relevant outcomes for patients hospitalized with community-acquired pneumonia.* Chest, 2009. **135**(6): p. 1572-9.

8. Christ-Crain, M., et al., *Free and total cortisol levels as predictors of severity and outcome in community-acquired pneumonia.* Am J Respir Crit Care Med, 2007. **176**(9): p. 913-20.

9. Menendez, R., et al., *Markers of treatment failure in hospitalised community acquired pneumonia.* Thorax, 2008. **63**(5): p. 447-52.

10. Martin-Loeches, I., et al., *Predicting treatment failure in patients with community acquired pneumonia: a case-control study.* Respir Res, 2014. **15**.

11. Zhydkov, A., et al., *Utility of procalcitonin, C-reactive protein and white blood cells alone and in combination for the prediction of clinical outcomes in community-acquired pneumonia.* Clinical Chemistry and Laboratory Medicine, 2015. **53**(4): p. 559-66.

12. Schuetz, P., et al., *Prohormones for prediction of adverse medical outcome in community-acquired pneumonia and lower respiratory tract infections.* Critical Care, 2010. **14**(3): p. R106.
