# Supplementary material for: Predictors of treatment failure for non-severe childhood pneumonia in developing countries – systematic literature review and expert survey – the first step towards a community focused mHealth risk-assessment tool?
Source: BMC Pediatr. 2015 Jul 9;15:74. doi: 10.1186/s12887-015-0392-x (PMC4496936; doi:10.1186/s12887-015-0392-x)
Supplement: Additional file 3: Expert panel interview materials. — ᅟ [file 12887_2015_392_MOESM3_ESM.pptx]

## Slide 1
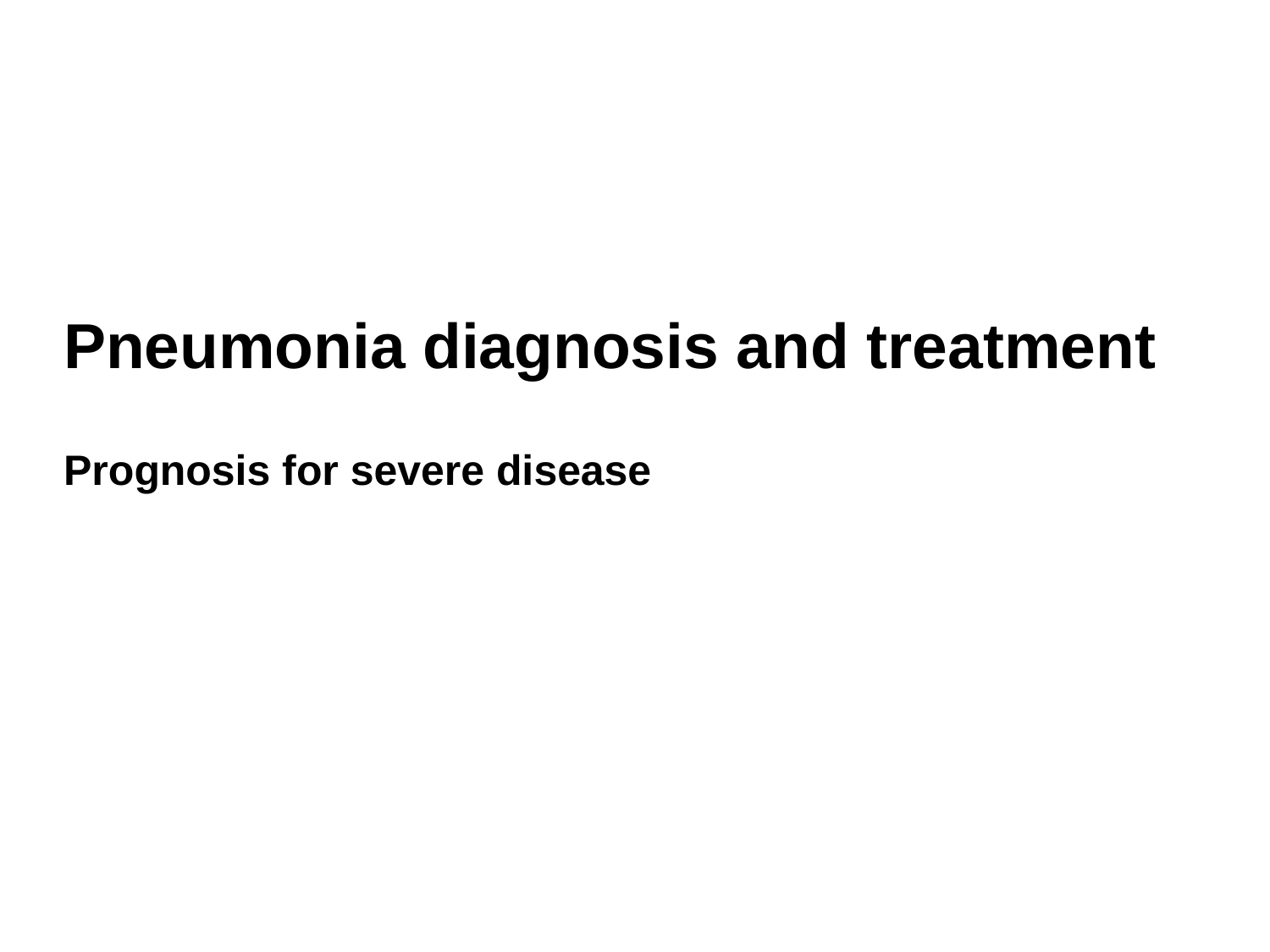

Pneumonia diagnosis and treatment
Prognosis for severe disease

## Slide 2
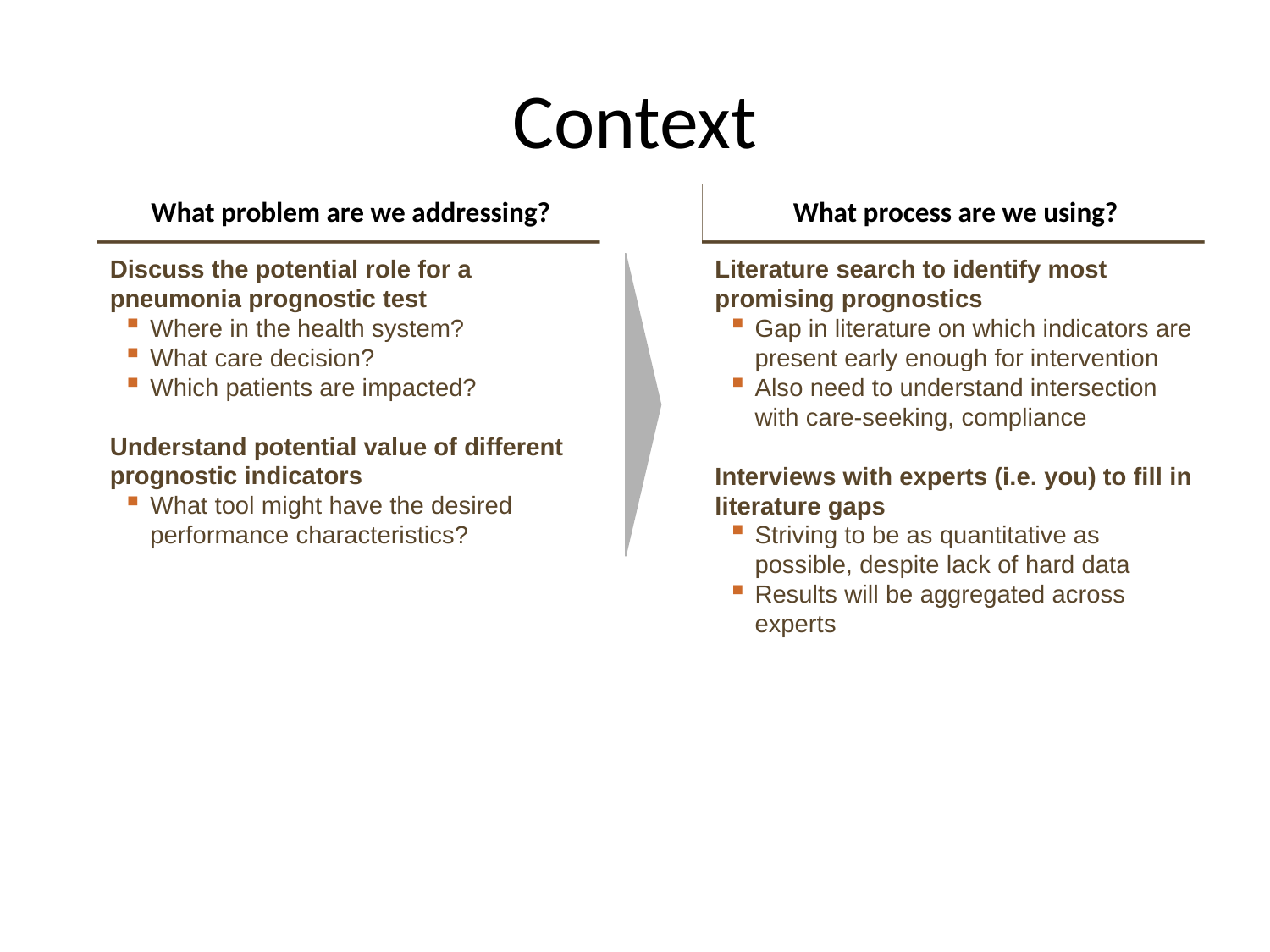

# Context
What problem are we addressing?
What process are we using?
Discuss the potential role for a pneumonia prognostic test
Where in the health system?
What care decision?
Which patients are impacted?
Understand potential value of different prognostic indicators
What tool might have the desired performance characteristics?
Literature search to identify most promising prognostics
Gap in literature on which indicators are present early enough for intervention
Also need to understand intersection with care-seeking, compliance
Interviews with experts (i.e. you) to fill in literature gaps
Striving to be as quantitative as possible, despite lack of hard data
Results will be aggregated across experts

## Slide 3
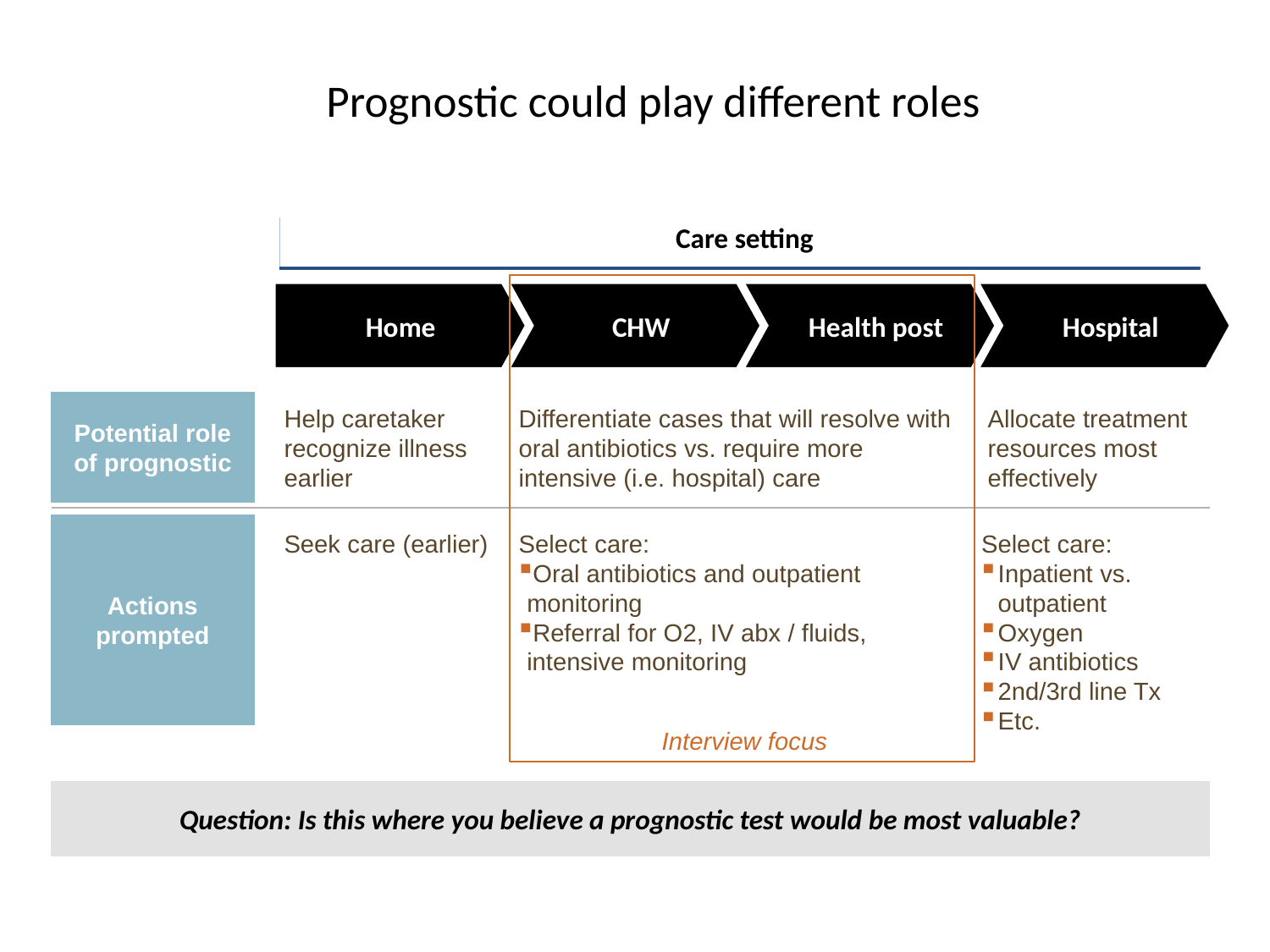

# Prognostic could play different roles
Care setting
 Interview focus
Home
CHW
Health post
Hospital
Potential role of prognostic
Help caretaker recognize illness earlier
Differentiate cases that will resolve with oral antibiotics vs. require more intensive (i.e. hospital) care
Allocate treatment resources most effectively
Actions prompted
Seek care (earlier)
Select care:
Oral antibiotics and outpatient monitoring
Referral for O2, IV abx / fluids, intensive monitoring
Select care:
Inpatient vs. outpatient
Oxygen
IV antibiotics
2nd/3rd line Tx
Etc.
Question: Is this where you believe a prognostic test would be most valuable?

## Slide 4
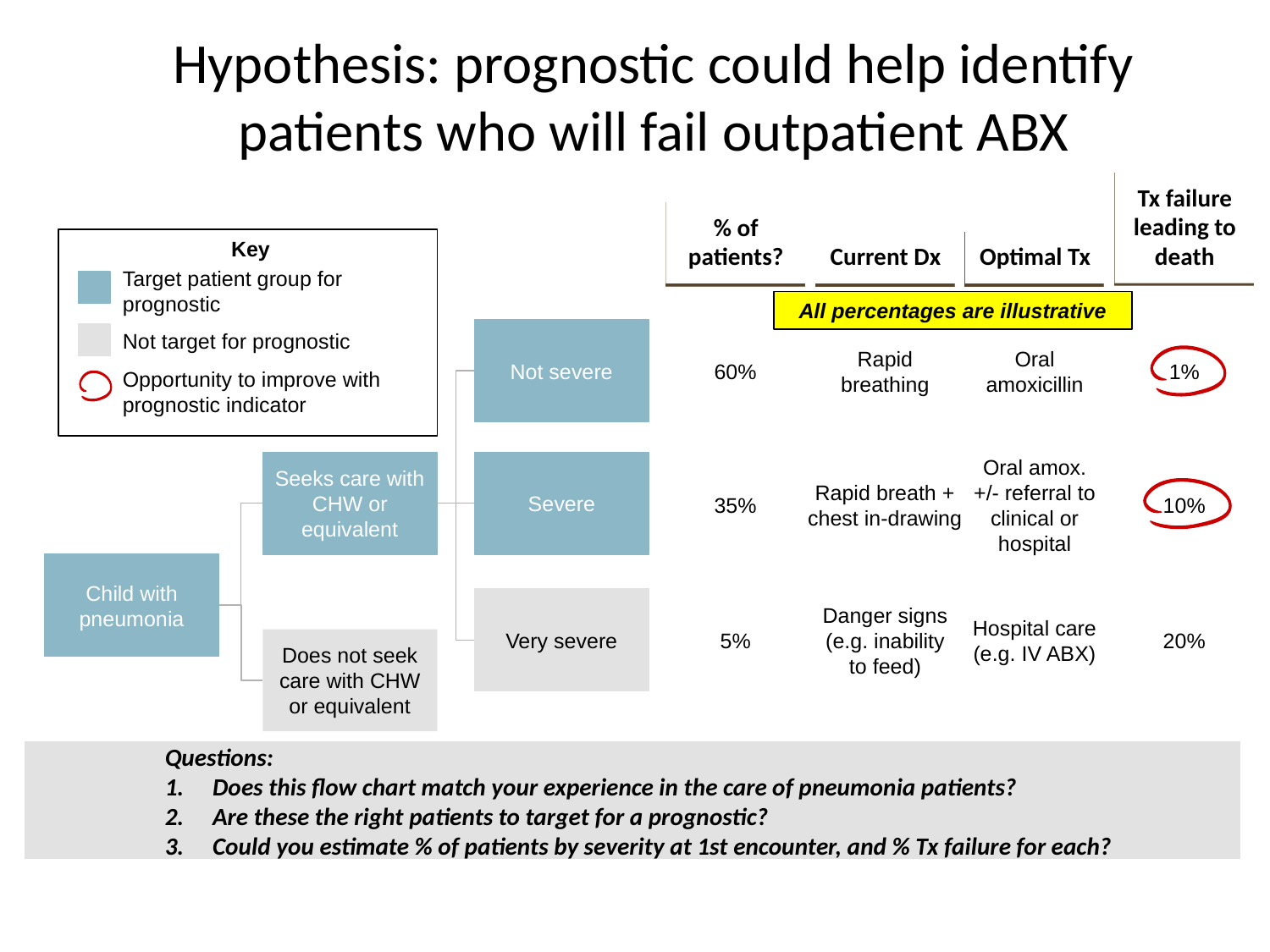

# Hypothesis: prognostic could help identify patients who will fail outpatient ABX
Tx failure leading to death
% of patients?
Current Dx
Optimal Tx
 Key
Target patient group for prognostic
All percentages are illustrative
Not target for prognostic
Not severe
60%
Rapid breathing
Oral amoxicillin
1%
Opportunity to improve with prognostic indicator
Seeks care with CHW or equivalent
Severe
35%
Rapid breath + chest in-drawing
Oral amox. +/- referral to clinical or hospital
10%
Child with pneumonia
Very severe
5%
Danger signs (e.g. inability to feed)
Hospital care (e.g. IV ABX)
20%
Does not seek care with CHW or equivalent
Questions:
Does this flow chart match your experience in the care of pneumonia patients?
Are these the right patients to target for a prognostic?
Could you estimate % of patients by severity at 1st encounter, and % Tx failure for each?

## Slide 5
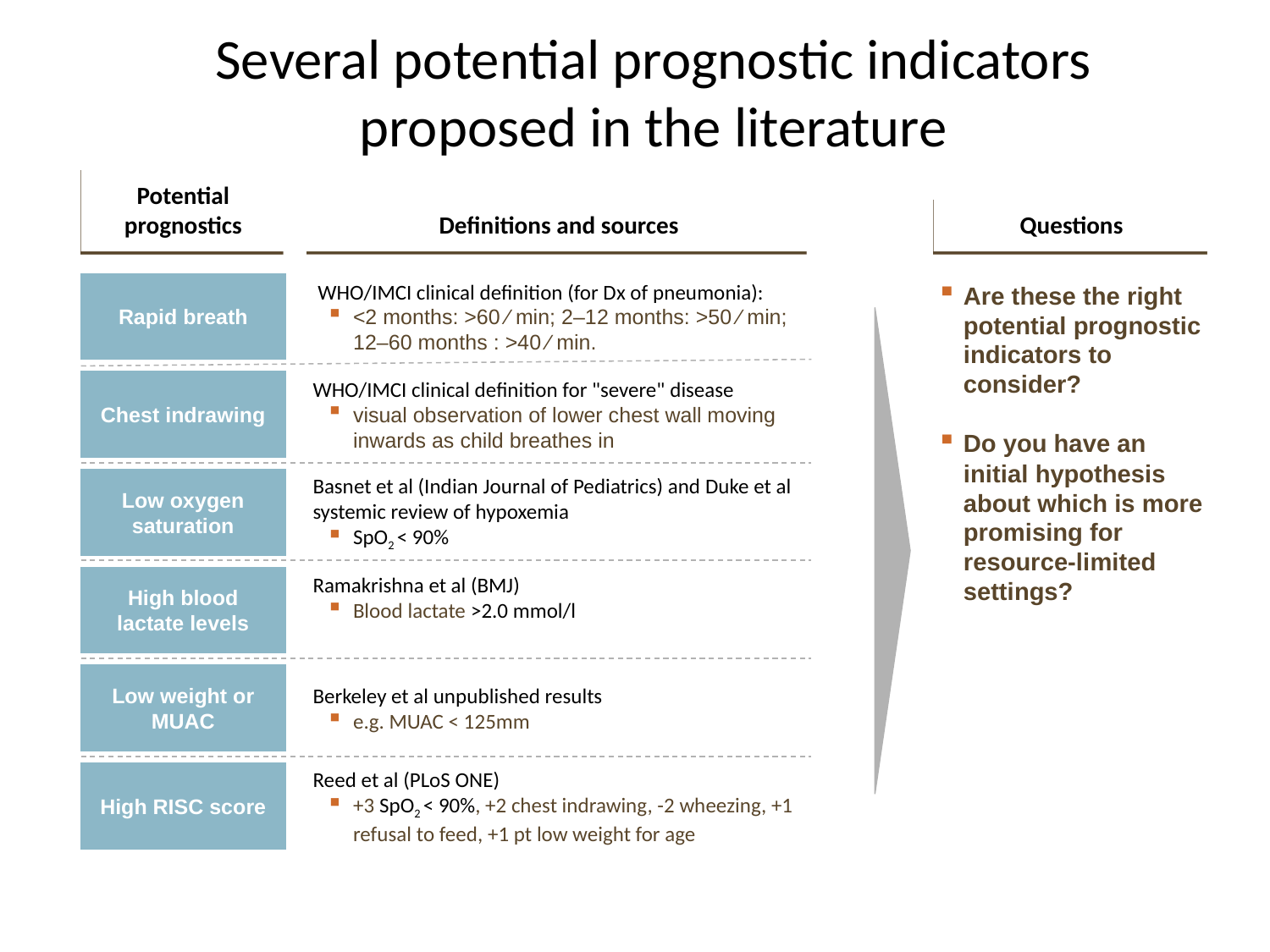

# Several potential prognostic indicators proposed in the literature
Potential prognostics
Definitions and sources
Questions
Rapid breath
 WHO/IMCI clinical definition (for Dx of pneumonia):
<2 months: >60 ⁄ min; 2–12 months: >50 ⁄ min; 12–60 months : >40 ⁄ min.
Are these the right potential prognostic indicators to consider?
Do you have an initial hypothesis about which is more promising for resource-limited settings?
Chest indrawing
WHO/IMCI clinical definition for "severe" disease
visual observation of lower chest wall moving inwards as child breathes in
Low oxygen saturation
Basnet et al (Indian Journal of Pediatrics) and Duke et al systemic review of hypoxemia
SpO2 < 90%
High blood lactate levels
Ramakrishna et al (BMJ)
Blood lactate >2.0 mmol/l
Low weight or MUAC
Berkeley et al unpublished results
e.g. MUAC < 125mm
High RISC score
Reed et al (PLoS ONE)
+3 SpO2 < 90%, +2 chest indrawing, -2 wheezing, +1 refusal to feed, +1 pt low weight for age

## Slide 6
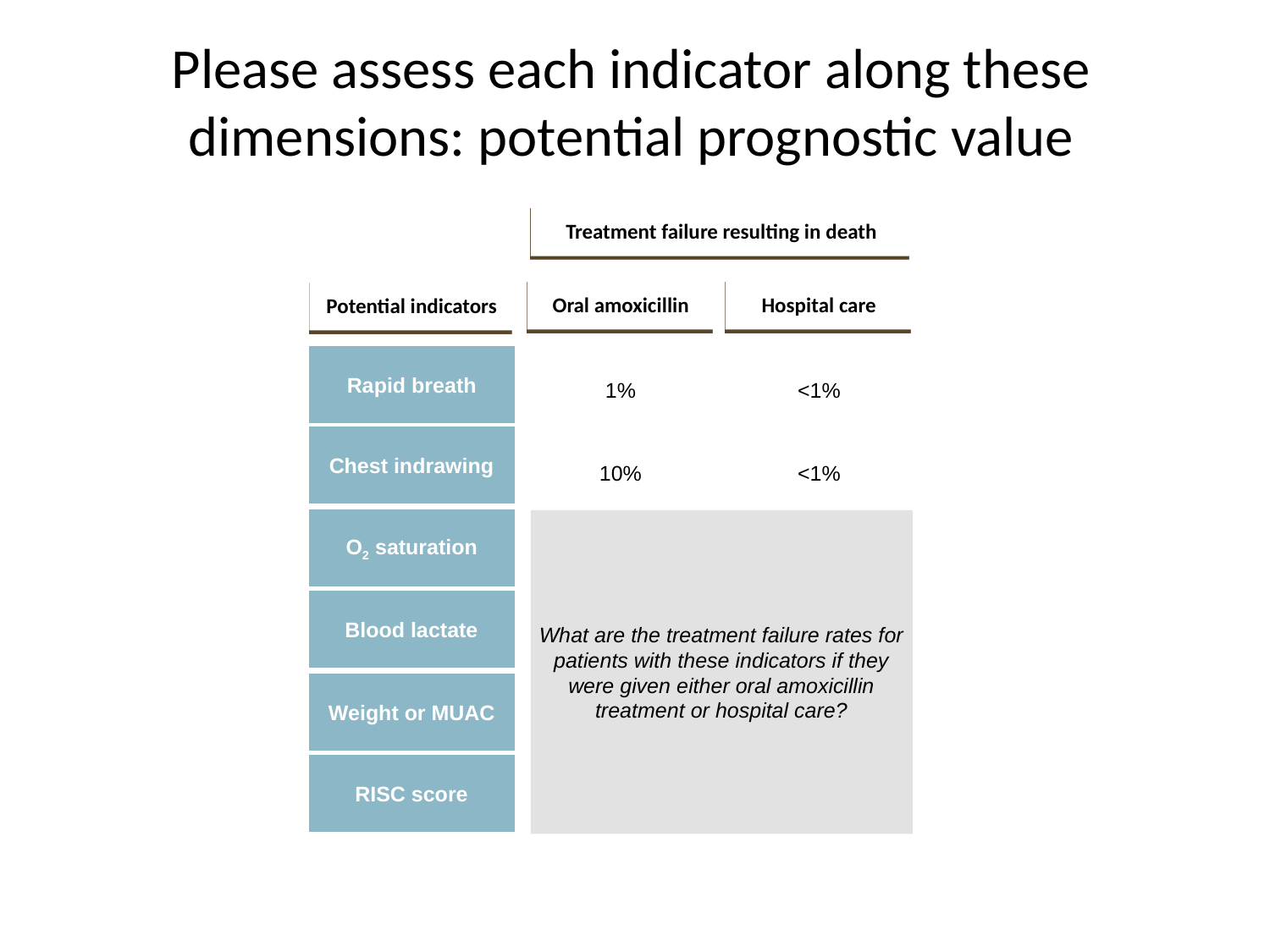

# Please assess each indicator along these dimensions: potential prognostic value
Treatment failure resulting in death
Oral amoxicillin
Potential indicators
Hospital care
Rapid breath
1%
<1%
Chest indrawing
10%
<1%
What are the treatment failure rates for patients with these indicators if they were given either oral amoxicillin treatment or hospital care?
O2 saturation
Blood lactate
Weight or MUAC
RISC score
